# Smartphone Self-Monitoring by Young Adolescents and Parents to Assess and Improve Family Functioning: Qualitative Feasibility Study

**DOI:** 10.2196/15777

**Published:** 2020-06-23

**Authors:** Dallas Swendeman, Stephanie Sumstine, Amber Brink, Deborah Mindry, Melissa Medich, Michael Russell

**Affiliations:** 1 Global Center for Children and Families, Department of Psychiatry and Biobehavioral Sciences David Geffen School of Medicine University of California Los Angeles, CA United States; 2 Department of Community Health Sciences Fielding School of Public Health University of California Los Angeles, CA United States; 3 Center for Culture and Health, Department of Psychiatry and Biobehavioral Sciences David Geffen School of Medicine University of California Los Angeles, CA United States; 4 Department of Family Medicine David Geffen School of Medicine University of California Los Angeles, CA United States; 5 The Methodology Center Department of Behavioral Health The Pennsylvania State University University Park, PA United States

**Keywords:** adolescents, parenting, conflict, self-monitoring, smartphones, mHealth, ecological momentary assessment, mobile phone

## Abstract

**Background:**

The natural integration of mobile phones into the daily routines of families provides novel opportunities to study and support family functioning and the quality of interactions between family members in real time.

**Objective:**

This study aimed to examine user experiences of feasibility, acceptability, and reactivity (ie, changes in awareness and behaviors) of using a smartphone app for self-monitoring of family functioning with 36 participants across 15 family dyads and triads of young adolescents aged 10 to 14 years and their parents.

**Methods:**

Participants were recruited from 2 family wellness centers in a middle-to-upper income shopping area and a low-income school site. Participants were instructed and prompted by alarms to complete ecological momentary assessments (EMAs) by using a smartphone app over 2 weeks 4 times daily (upon waking in the morning, afternoon, early evening, and end of day at bedtime). The domains assessed included parental monitoring and positive parenting, parent involvement and discipline, parent-child conflict and resolution, positive interactions and support, positive and negative affect, sleep, stress, family meals, and general child and family functioning. Qualitative interviews assessed user experiences generally and with prompts for positive and negative feedback.

**Results:**

The participants were primarily white and Latino of mixed-income- and education levels. Children were aged 10 to 14 years, and parents had a mean age of 45 years (range 37-50). EMA response rates were high (95% to over 100%), likely because of cash incentives for EMA completion, engaging content per user feedback, and motivated sample from recruitment sites focused on social-emotional programs for family wellness. Some participants responded for up to 19 days, consistent with some user experience interview feedback of desires to continue participation for up to 3 or 4 weeks. Over 80% (25/31) of participants reported increased awareness of their families’ daily routines and functioning of their families. Most also reported positive behavior changes in the following domains: decision making, parental monitoring, quantity and quality of time together, communication, self-regulation of stress and conflict, discipline, and sleep.

**Conclusions:**

The results of this study support the feasibility and acceptability of using smartphone EMA by young adolescents and parents for assessing and self-monitoring family daily routines and interactions. The findings also suggest that smartphone self-monitoring may be a useful tool to support improvement in family functioning through functions of reflection on antecedents and consequences of situations, prompting positive and negative alternatives, seeding goals, and reinforcement by self-tracking for self-correction and self-rewards. Future studies should include larger samples with more diverse and higher-risk populations, longer study durations, the inclusion of passive phone sensors and peripheral biometric devices, and integration with counseling and parenting interventions and programs.

## Introduction

### Background

Research demonstrates that family processes in daily routines and settings have significant impacts on children’s development and well-being [1-4]. The feelings, actions, and interpersonal interactions of individuals are structured by daily routines that influence the household and family. Thus, family routines provide a bridge between individual and systemic levels of the multilevel family system [3,4]. Key factors in daily family routines include parent-child communication and family interactions. Lack of parent-child communication has been associated with low life satisfaction for adolescents [5,6]. In contrast, parent-child conflict and perceived lack of support have been associated with negative psychological, social, and health risks for children (ie, depression) [7,8]. Conversely, positive family interactions have been linked to decreases in internalizing emotional distress [9,10]. Emotional states such as affect, conflict, and stress can also be transmitted between parents and their children [9,11,12]. Family stress can also negatively impact peer relationships of adolescents and school domains [13]. Fostering positive interactions, communication, support, and conflict resolution within families may better protect families from maladaptive outcomes such as depression, behavioral and school problems, lower self-esteem, and poor social skills [8].

Engaging families in therapeutic activities addressing family processes in real time during daily routines is a persistent challenge in interventions and research [14]. The broad proliferation of mobile phones creates novel opportunities for interventions and research modalities that are integrated into daily routines and are widely scalable. Self-monitoring is one strategy that can be easily implemented via smartphones. Early research on self-monitoring recognized reactivity to self-assessments as a means to support self-regulation and behavior change through feedback and goal-setting processes [15-18]. One form of self-monitoring is daily diaries and ecological momentary assessment (EMA). EMAs are repeated self-reports conducted multiple times throughout a day to assess behaviors, attitudes, states, and experiences in real time, in the natural environments of subjects [19]. EMA has greater ecological validity, fewer recall biases compared with observational or global questionnaire methods, and the capacity to elucidate within- and between-person processes and temporal dynamics [19]. For example, utilizing EMA in family interventions can allow researchers to examine the relationships between intrapersonal processes (ie, mood), interpersonal processes (ie, supportive or hostile exchanges), and broad family-level contexts (ie, family conflict, cohesion) that may address more complex and nuanced questions about sequential processes that influence behavior and affect in the daily lives of individuals [20].

EMA and diary methods have been used to study family experience in daily routines across multiple domains such as parent-child interactions [8], family relationships [21], family conflict [13], and stress [10,22]. Notably, the intensive nature of daily diaries and EMA may result in reactivity (ie, changes in awareness and behavior, particularly in populations motivated to change [19,23]). This is a methodological nuisance of basic behavioral research but presents a potential opportunity for self-monitoring as an ecological momentary intervention [23]. The little research done previously on reactivity has favored minimizing reactivity and its related effects [19], including in family research [24]. In general, EMA and diary research does not address reactivity routinely or robustly [23]. Most important to family research, EMA allows for real-time collection of data from multiple informants (ie, multiple family members) who often share the same natural environments, while also experiencing similar *events* (ie, family meals, arguments) [20]. Using EMA as an assessment tool in families allows different perceptions of the same experiences and the ability to identify discrepancies in perception.

### Objectives

This paper examines the user experiences of families on feasibility, acceptability, and perceived benefits of self-monitoring and reactivity to smartphone EMA and daily diaries for assessment, self-monitoring, and as a potential tool for intervention to seed and support behavior change.

## Methods

### Sample and Recruitment

This study enrolled 36 participants across 15 families consisting of 15 children in 9 family dyads (all mother and child) and 6 family triads (mother, father, and child). The child participants included 6 boys and 9 girls aged between 10 and 14 years. Participants were recruited through a family wellness center’s e-mail newsletter and website. The family wellness centers, funded by the Robert Wood Johnson Foundation, provided social and emotional learning and physical activities in a metropolitan US community at a shopping area marketplace-based site (average income of US $67,000), and at a middle school located in a low-income neighborhood (average income of US $27,000) serving primarily Central American and Korean immigrant populations. Over one-third (n=6) of participant families came from the low-income site, 5 of which were Latino. Only 1 family from the middle-income site was Latino, whereas the rest were white, Asian, or African American. Prospective participants were informed that this was a pilot study to develop and test a smartphone app designed to enrich our understanding of ways to improve daily family routines and well-being.

Families who called the study contact were screened for the following eligibility criteria: parent and child coresided for some portion of the 2-week study period, at least one parent agreed to participate, the child was aged 10 to 14 years and gave assent to participate, and participants were fluent in English. Families with multiple children had the option to enroll again to participate with another child in the family (2 families exercised this option). Participants signed informed consent forms according to the university’s institutional review board–approved protocols.

### Procedures

After consent at an in-person meeting, participants were issued a smartphone (Samsung Galaxy S) for the study on which they completed EMA and daily diary surveys 4 times per day for 2 weeks. The study coordinator gave participants a brief training on how to use the smartphone and a step-by-step instructional manual on how to use the smartphone app platform to complete EMA surveys. All participants were given the study coordinator’s phone number in case they had questions or experienced any difficulties while using the smartphone. At the end of the EMA period, qualitative interviews lasting approximately 40 min assessed the user experiences of parents and children, reactions to using the smartphone app, the obtrusiveness of the monitoring, any technical problems they encountered, relevance and usefulness of the EMA and diary questions, and perceived effects of study participation on them and their family. The semistructured interview guide first queried for general feedback and experiences, followed by prompts for “what was useful or helpful?” then “what was not helpful, or annoying?” and finally, suggestions for changes or improvements to the protocol and app. Participants also completed web-based questionnaires on demographic characteristics and family functioning at the start and end of their study participation. Participants received gift cards valued up to US $150 for completion of the different components of the study.

EMA and diary data were collected using Ohmage, an open-source mobile survey app supported by a web platform that supports the collection, storage, analysis, and visualization of EMA or self-monitoring data streams. Ohmage is a feature-rich and extensible platform that facilitates the collection of multidimensional, heterogeneous, and complex personal data streams. The software was programmed using time-based reminders to display question sequences and response choices on the smartphone screen. EMA survey responses were automatically timestamped, geotagged, and linked to the participant’s assigned study identifier used as their login ID. Web interfaces were available for researchers to access and view participant data. The Ohmage user interface was designed based on feedback from behavioral and technology researchers focusing on group participants and end users of the system [25].

Participants were prompted to respond 4 times daily to EMA/diary surveys on the following domains: parental monitoring and positive parenting, parent involvement and discipline, parent-child conflict and resolution, positive interactions and support, positive and negative affect, sleep, stress, family meals, and general child and family functioning. Although many family assessment tools are widely available to researchers, clinicians, and families, none directly measure daily routines in real time. EMA/diary domains were chosen based on systematic reviews of standardized family functioning measures [26-29], which consistently assess communication, conflict, problem solving, cohesion or bonding, affect or emotion, organization, or regulation (eg, roles, rules, leadership, monitoring, and stress; see Table 1). EMA/diary questions were adapted from retrospective or global self-reported family measures. Domain and measure selection decisions were also informed by their use in intervention research with high-risk adolescents and the desire to balance with domains linked to resilience and wellness. Table 1 shows the EMA/diary domains and global/retrospective self-report measures that were adapted for EMA format. EMA/diary question contents are available as Multimedia Appendices 1-3.

The timing of the EMA vibration/ring prompts was scheduled by the participants and the study coordinator at times convenient for their individual schedules as follows: (1) morning upon awakening, (2) before school/work, (3) between the end of the school or work day and dinner, and (4) before bedtime. Upon hearing the reminder, participants were instructed to stop their current activity and complete a short (less than 5 min) EMA. Families received 1 phone call on the third day of the EMA period from the study coordinator to inquire about technical problems with the smartphone and app and answer any other study questions.

**Table 1:** Smartphone ecological momentary assessment and daily diary measures, sources, and schedule.

Domains	Measures and sources (parent and child, unless noted)	Schedule
Sleep	Number of hours and subjective quality (1=very poor, 5=very well)	Wake-up only
Stress	Single rating of current stress level (1=not, 5=very)	All
Affect and mood	Positive and negative affect schedule [30] and personal affect measure [31]	All
Monitoring/positive parenting	Stattin and Kerr parental monitoring questionnaire [32,33]	3 × (not wake-up)
Parent involvement and inconsistent discipline	Alabama parenting questionnaire [34,35]	3 × (not wake-up)
Parent-child conflict	Issues checklist [36,37] and network of relationships inventory (child) [38]	3 × (not wake-up) end of day only
Conflict resolution	Conflict tactics scale, resolution subscale [39]	3 × (not wake-up)
Positive interactions	Network of relationship inventory, companionship subscale [40]	End of day only
Family meals	Who do you eat with and doing other activities?	End of day only
Overall functioning	Outcome rating scale and child outcome rating scale [41]	End of day only

### Data Analysis

Descriptive analyses for demographic characteristics were conducted using simple frequency distribution statistics in Stata 15.1 (StataCorp). The qualitative user-experience interviews were audio recorded and transcribed verbatim. Of the 36 participants, 31 had audio recorded interviews available for transcription (5 audio files were inadvertently erased before transcription), and transcripts were redacted to remove personal identifying information and uploaded to the Dedoose web-based mixed methods analysis platform (version 4.5.91, Sociocultural Research Consultants 2013). A grounded theory inductive approach was used to code the data to identify key themes that emerged from the data [42,43]. The coding scheme was developed by the lead anthropologist with 2 research assistants. The research assistants engaged in initial discussion around substantive codes emerging from the data and analytic categories that evolved into tangible themes; the generated codes were organized into broader, more conceptual themes. The lead anthropologist reviewed all themes identified by the research assistants for the discrepant cases. The codes were shared with the research team and revised over several iterations. Codes and excerpts were retained for analysis when there was agreement between the coders and authors.

## Results

Children were on average aged 12 years (SD 1.44), mothers were 46.25 years (SD 3.81), and fathers were 40.33 years (SD 3.51). Approximately half were white (n=20), one-third were Latino (n=11), 9% (3/35) were Asian, and 3% (1/35) were black. Tables 2 and 3 present more demographic results for children and parents, respectively. Response rates were high overall, including some participants who completed more EMAs than scheduled (prompted), either by reporting for more than 14 days or reporting more on some days to compensate for missed EMAs (typically for the previous day). Overall, the response rate excluding more than 4 EMAs in a day and more than fourteen days of reporting (ie, the on-time and per protocol response rate) was 96.2% (1941/2016), with children slightly lower at 95.1% (799/840) and parents slightly higher at 97.1% (1142/1176). Overall, 69% (25/36) of the participants had 100% or greater response rates; 60% of the children and 76% of the parents. The lowest response rate among children was 70% (39/56) and 79% (44/56) among parents. A total of 6 parents and 3 children responded for 16 to 19 days. In terms of missed EMAs, children tended to miss the morning and noontime EMAs, whereas parents tended to miss the late afternoon/early evening EMAs followed by the morning EMAs.

Qualitative results from the analysis of user-experience interviews are presented below based on 2 broad code themes and several subthemes that emerged from the data. The first broad code theme was feasibility, acceptability, and suggestions for the future, with the subcodes desire for feedback, seasonality, technical problems/challenges, survey burden, timing and frequency (weekends and duration), and global/recall web surveys. The second broad code theme was self-reflection, awareness, and seeds of change, with the subcodes decision making, parental monitoring, quality and quantity of time spent together, communication, self-regulation of stress and conflict, discipline, rewards and punishments, and sleep.

**Table 2:** Demographic characteristics of children at baseline (N=15).

Characteristic		Participants, n (%)
**Age (years)**		
	10	3 (20)
	11	2 (13)
	12	4 (27)
	13	3 (20)
	14	3 (20)
**Grade**		
	4	1 (7)
	5	2 (13)
	6	2 (13)
	7	4 (27)
	8	2 (13)
	9	4 (27)
**Ethnicity**		
	White	10 (67)
	Latino or Hispanic	4 (27)
	Black or African American	0 (0)
	American Indian or Native American	0 (0)
	Asian or Pacific Islander	2 (13)
	Other	0 (0)

**Table 3:** Demographic characteristics of parents at baseline (N=20).

Characteristic		Participants
**Age (years), mean (range)**		
	Informed	45 (37-50)
	Missing	10 (50)
**Gender, n (%)**		
	Female	14 (70)
	Male	6 (30)
**Marital status, n (%)**		
	Married	18 (90)
	Separated	1 (5)
	Living with a partner	1 (5)
**Ethnicity, n (%)**		
	White	10 (50)
	Latino or Hispanic	7 (35)
	Black or African-American	1 (5)
	Asian or Pacific Islander	2 (10)
**Highest level of education, n (%)**		
	11th grade	1 (5)
	12th grade	2 (10)
	1-year trade, college, or university	1 (5)
	3-year trade, college, or university	2 (10)
	4-year trade, college, or university	4 (20)
	Graduate education	9 (45)
**Total family income (before taxes or other deductions; US $), n (%)**		
	10,000 to 29,999	2 (10)
	30,000 to 49,999	2 (10)
	50,000 to 69,999	4 (20)
	70,000 to 89,999	1 (5)
	90,000 to 99,999	1 (5)
	100,000 and over	8 (40)
	Missing	2 (10)
**How difficult is it for you to live on your total household income right now?, n (%)**		
	Not at all difficult	9 (45)
	A little difficult	6 (30)
	Somewhat difficult	3 (15)
	Very difficult	2 (10)
**Do you have a spouse or partner with whom you share parenting responsibilities (for this child)?, n (%)**		
	Yes	19 (95)
	No	0 (0)
	Missing	1 (5)

### Feasibility, Acceptability, and Suggestions for the Future

Participants found the smartphone self-monitoring feasible and acceptable and provided feedback for changes and improvements. Most participants reported enjoying their participation in the study:

Oh, it was, it was a really nice experience… it’s like not every day you can talk to somebody about all the stuff they ask you in the app, in the surveys. Yeah… it was kind of a little fun to do ‘cause I was like okay, just waiting to do it, you know? I’ve [sic] taken another kind of like testy thing. I always liked taking them and I hardly took ‘em, so this was like, “Finally. Yay! I can do another one.”

male child (14 years), family 5

The same participant also noted the convenience of smartphone surveys in comparison to web-based surveys on a computer:

I think it’s better, kind of, to take the phone surveys because it’s kind of way more convenient for people that are busy, ‘cause they’re shorter and you can take ‘em wherever, anywhere you go. …Web surveys, you have to be on a computer, and you have to have time. And, so it may not be useful or good for people with a busy schedule.

male child, 14 years, family 5

### Technical Problems and Challenges

Participants reported a few minor technical problems associated with the mobile phone, such as slow uploading of data or having to power the phone off and on to upload data. Some participants reported problems with the app freezing or force closing when they were trying to complete a survey. A few participants reported that they were not receiving reminders (alarms) to complete the surveys after a period.

### Seasonality

Some participants noted that the survey questions needed to be geared toward the time of year. For example, for a number of participants who participated during the summer school break, questions about school and homework were inappropriate:

There were parts that didn’t seem to apply because it seemed like the survey was sort of designed to assess children when they’re in school. So, since it’s summer, sometimes, you know, the questions didn’t seem to apply, and then particularly with the, the final assessment, there was a lot of stuff about school. So, some things we didn’t know how to respond to…

mother, family 6

### Survey Burden: Timing and Frequency

With regard to the smartphone EMA, 10 participants reported finding the end of day bedtime survey burdensome because it was long, and several noted being tired:

The only thing that I would say is, I thought the evening one was really long. And, I think that the evening one should have been short. I think the length should have switched…[the] afternoon could have been the longer one, and the evening one could have been shorter, because I… I took it at like ten or whatever, and I was already really tired and I didn’t really want to take a really long survey.

male child, 13 years, family 8

Some noted the value of the longer end of the day survey. For example, one mother noted the end of the day survey allowed her to reflect on her day:

Yeah, I mean, obviously, the last one of the day was longer, so that was the only thing. Like if I wasn’t taking it at a decent hour, and I was super tired… that was the only, the only thing about that. But, I mean, I think that’s when you have to have the long one because that’s when you’re really reflecting on everything that happened that day.

mother, family 8

Some participants also felt that there were just too many surveys to do each day:

Well, I think having to answer four times a day was too much. I would have preferred like breakfast, lunch and dinner. The wake up and the, you know, so that was a little annoying, just a little bit. And then, after so many days, it was like, “Oh my God. Did I do this?”

mother, family 5

Some participants noted the value of multiple surveys over the course of the day for transient states as a means of checking in and reflecting:

I think it was good timing ‘cause, you know, in the morning, when you wake up, you have a different mood, and then in the afternoon, you have a different, and then you have different like every two hours or three.

female child, 12 years, family 13

### Weekends

A few participants noted that it was difficult to complete surveys on weekends when daily activities and schedules are less routine:

I know that some days, it was more of a challenge to complete them kind of in the actual set time, especially on the weekends is really hard because by not being sort of on that same schedule.

mother, family 1

### Duration

Most participants found the 2-week study period a good time period:

No, I think that’s perfect [two weeks]. I think that was really, that was really good. It, it was just long enough to where it became a habit and you’re makin’ sure you aren’t forgetting it. And then, but then towards the, near the end, you can just tell for, lookin’ forward to just bein’ finished ‘cause it does become a burden…

father, family 1

However, a few participants suggested a longer duration, particularly to support behavior change:

I, to be honest with you, I found myself wanting to continue….Two weeks would be a minimum. A month would be a maximum. You know, for me in my imaginary world - three weeks would be ideal, just because it, it gives you that, that first week - it was more about, “Hey,” at least for me, it was like, “Okay. Let’s just follow the behavior.” The second week, it was like, “Okay. How am I gonna improve the behavior.” But now, I don’t have a chance to see if, to implement it, and to see how, how it’s going. So, I was like, “Hey, okay. We have to give the phones back, alright.” (laughter) So, that, that left, that left me wanting more.

mother, family 2

Probably about a week longer, but any more than that, I think it would have gotten like really repetitive and the answers would have all been the same.

female child, 13 years, family 7

### Desire for Feedback

Participants noted that they would have liked the feedback from their participation in the study to know the findings from the survey and how these could help them and their families improve their relationships. Parents were very interested in getting more feedback from the study to improve their parenting skills and strategies. For example:

I think, eventually, if this was gonna be something more, intervention oriented, it would be interesting, that kind of assessment, even if it’s something like, sort of the average, an average for the family and, and how that changes depending on, sleep or, I don’t know. Changes in communication. I’m kind of more interested in just our overall picture as a family. And, where we need to improve…and I could sort of pick up on that based on the things that we were being asked.

mother, family 6

### Self-Reflection, Awareness, and Seeds of Change

Over 80% (25/31) of the participants reported increased awareness of their relationship dynamics with their child/parent, their own behavior, or their communication styles. Of the 5 participants who did not report changes in awareness of their family routines, 4 were children. For many participants, study participation provided novel opportunities to reflect on their family routines in general:

I thought it was a very good exercise in terms of just self-reflection on things you do every day… you get on automatic pilot sometimes. You don’t stop and really consider, “How, how did that go?” (laughter) So, I like that aspect because it sort of forced me to check in with myself and see how things were going.

mother, family 1

It’s basically somethin’ out of the ordinary, (laughter) to kind of express your feelings throughout the day. …But, to me, it was a productive kind of thing because, when you’re thinkin’ about what’s goin’ on for the day, you were able to reply too. I thought I was just holding it in, letting it out, you know, at the end of the week or somethin’.

father, family 5

One child noted increased awareness of his lack of contribution to household chores from 1 of the question prompts:

This question in particular, “I’m not as responsible as I should be.” I answered that, I’m like, “You know what? I, I think I agree with that.” And, that made me feel kind of bad. So, from, from that point on I started doing, now I unload the dishwasher.

male child, 12 years, family 11

These themes of reflection-seeding behavior change are represented throughout participants’ feedback on their experiences in more specific domains (described below).

### Decision Making

Several parents reflected on decision making in the family as a result of self-monitoring. For example, 1 noted:

Well, it made me think about how we decide is this something we’re gonna give [daughter] input into? Or, is this something we just decide because, you know, we’re the parents? It kind of made me think, sometimes do we give her too much input? (laughter)

mother, family 10

I see. You were becoming aware of how family decisions were…?

interviewer

Yeah, how family decisions were made I really wish my husband could have taken it. It would be good for him too. And, it was just kind of interesting to think about…

mother, family 10

### Parental Monitoring

Children, in particular, described increased awareness of parental monitoring. For example, 1 child noted:

I realized my mom stays out of my life and I stay out of hers. (laughter)… ‘Cause, I don’t, I tell my mom some things….And, she never really asks what I’m doing. Like she, she pretty much knows like “he was outside,” or “he was on the computer.” She doesn’t know specifically what I’m doing, but she’s always got a vague idea, so….I think that’s what I realized the most…some of the different questions like, “Did your parent know where you were at night?” …It made me realize, yeah, she pretty much always knows where I am, even when I’m not using my phone. So, I’m like, “I guess we are pretty connected.”

male child, 12 years, family 11

### Quality and Quantity of Time Spent Together

Participants reflected that the quantity and quality of time spent together were both important. One parent reported becoming aware of how seldom her family ate together:

It makes you think about stuff. Like, are we talking? Are we eating together? And, it’s kind of embarrassing like, “Ugh,” we eat alone, or so and so ate with so and so. You know? That was cool for us as a family to think, “Oh my gosh. We don’t eat together.”

mother, family 5

In addition to self-reflective functions, families noted how EMA/diary self-monitoring helped them to be more accountable or consistent with their values or goals for their families. For example, 2 parents indicated an element of accountability from self-monitoring in regard to time with family:

The process and the idea behind it, I thought was actually really good, ‘cause it helps you be more conscious about spending time with your child and in, especially ‘cause you know you’re gonna be reporting it later, so you want to make sure you have some good things to put in there, as opposed to bad. So, I think it helps people be conscious of that relationship.

father, family 1

This father noted how the anticipation of reporting influenced his behavior. One mother noted more direct reminder functions:

But… it does make you think that maybe you should… just be more in touch, communicate more. You know, one thing that struck me was… the leisure time, spending fun time with your child…I’m working full-time, I’m not gonna have that, but on the weekend, you know? … I think it does sort of, at least for me, remind me that I do need to spend fun time with my child….

mother, family 6

Her daughter also noted becoming aware of needing to spend more time with her parents and considering a change in her routine:

I’d think back on like how I reacted to some things or the interaction I had with my parents. Like maybe I should be out in the house more, not in my room.

female child, 14 years, family 6

### Communication

Participants noted reflection, awareness, and some changes in patterns of communication in the family, including themes of limited time, positive or negative tone, praise or critique, and openness. For example, a mother stated:

I liked it [self-monitoring app]. It actually made me more aware of how my daughter and I communicate, and when we communicate.

mother, family 10

Another mother noted how she became aware of how little she communicated with her child:

But, in terms of the way I interact with my son, it was good because I didn’t realize how little I actually communicated with him. So, in some ways, I felt good about myself because I felt like, “Oh, well, I think I really trust my son because I don’t feel a need to constantly see what he’s doing or talk to him.” But then on the other hand, I thought, “Wow, two weeks has gone by and, really, we didn’t make time to talk.” So, it just made me a lot more aware.

mother, family 11

The son in this family also noted reactivity to self-monitoring and becoming aware of limited communication with his mother and that most often it involved giving him instructions:

I realized, I don’t spend that much time communicating with her. Like it’ll be like 10 minutes here, 20 minutes there, a minute there. Usually, it’s just we talk for a second and say like, “You gotta do this.” I’m like, “Okay.” And then I go do that, and that’s pretty much it.

male child, 12 years, family 11

Other families noted that self-monitoring reactivity led to more frequent communication:

I ended up speaking more to them - to my wife and my, my son, my other son too. Trying to eat dinner with them, trying to make more family time, you know?

father, family 5

A few participants noted increased awareness of needing to focus on positive communication and not just negative issues, and making changes based on this awareness:

I don’t think that either S. [father] or I are big complimenters and,…it tends to run more negatively, which I think when you really think about it, you go, “Oh, that’s not, that’s not really how I want to be, and that’s not how I feel, really.” It’s just trying to keep him afloat and making progress all the time, that you sort of get in these grooves. And so, I do think it was helpful, like I tried to… compliment him when he did something well.

mother, family 8

One father noted how the EMA questions also functioned as reminders for him:

I guess you don’t always tell your kids that they’re doin’ a good job. Like I, after the surveys, I started makin’ a point to tell him he was doin’ a good job. (laughter) So, I guess that that’s probably a good thing that the surveys tell you, is to remind you to remind, to let your kids know when they’re doing well or not.

father, family 1

Another parent noted how reactivity to self-monitoring functioned by modeling questions that deepen levels of communication with her son around the domains assessed:

So, there is that element of both of us are doing these surveys and then, for me, part of it was, “Okay, I want to start asking you these questions since I don’t really ask you these questions.” So, I would actually ask him more about the ins and outs of his day more, and then he would talk to me more about it ‘cause I was asking him for this information. …So, in a way, I was getting more information from him, and we were discussing more between the two of us than we would, normally, if I wasn’t doing the survey.

mother, family 3

Similarly, her son reflected that their improved communication made him feel closer to his parent:

I enjoyed getting closer to my mom throughout the study because she’d always ask me questions that she wouldn’t really normally ask me, and we got a little closer through that.

male child, 13 years, family 3

### Self-Regulation of Stress and Conflict

EMA self-monitoring was also reported to support the self-regulation of stress and staying calm, including during conflicts and their resolution. For example, 1 parent noted that self-monitoring helped her focus on trying to stay calm when interacting with her child:

Well, I tried to become more of being calm (laughter) and not yelling. And, that was really it. …I mean, obviously, if you’re tired or you’re, somethin’ else is going on, it’s hard to do that. But, when I was in a relaxed state, it made me mindful of, “Okay, when she does something that annoys you, just be calm about it, and try and work through it.” It doesn’t always happen. But, it did make me more cognizant to try and just be more patient and talk with her - depending on what it was.

mother, family 10

Similarly, a child noted that self-monitoring helped her and her mother identify what was making them angry and enabled them to resolve conflict:

When my mom and I would argue, and we would put it down [in the app], …we were like, “oh, we’re actually mad about something,” and then we would start thinking about like…Actually think about what happened.…and then we would talk and apologize.

female child, 12 years, family 2

And then you would talk and apologize. Is that something you did before?

interviewer

Not really because we wouldn’t think about it, we’d just get mad (laughter).

female child, 12 years, family 2

One child became aware that she could not remember why she was angry with her parent:

I think before I took the survey, I just wouldn’t think about why I was mad at her. I’d just be so mad. But then when I sat down and took the survey, and it was sayin’ what was it about - I was like, “Wait. I don’t even remember, anymore, what it was about.”

female child, 10 years, family 9

One mother noted that the EMA self-monitoring helped her communicate more calmly with her child when they were in conflict by reflecting on and using conflict resolution strategies represented in the EMA response options:

Well, there’s a certain question, for example, about, “Did you try to speak to your child calmly?”…The first time that I’m reading through them, I was like, “I don’t know. Did I even try? (laughter) Did I just start yelling? Did I?” It…literally had me stop and, and take a step back and remember the whole scenario. You know, literally picture by picture, and break it down. And then, I caught myself, it’s like, “I didn’t even try.” …and so after that, it was like, “Okay, let me try to speak to her calmly. Let me try and explain, you know, why the chair is yellow, and why the sky is blue.” (laughter) ….And, towards the end of these last two weeks, I wasn’t trying, I was speaking to her calmly.

mother, family 2

This mother also noticed her child using more positive reinforcement in her communication with a sibling, based on her initial reactivity to EMA and then her daughter modeling the behavior:

I noticed that she started it with her sister with the, “Hey, Sophie, I, thank you for helping me do the dishes for mom.” You know, something like that out of the blue. Or, she had to throw the trash out and, “Oh, Sophie - thanks for taking the trash out. I, I really appreciate that.”…It was a change. It was an actual, positive change, and it was after she caught on to what I was doing.

mother, family 2

### Discipline, Rewards, and Punishments

Although parents were generally not very comfortable talking about disciplining their children, many discussed becoming more aware of patterns of discipline or rules in their families. Some parents were more comfortable talking about rewarding or praising their children and were consciously working on improving their positive reinforcement of their children’s behavior. For example, a mother spoke about becoming more aware of limits and ground rules:

You know, and, and maybe because of the older one, she’s testing limits. You know, and it’s like, “Okay, but these are the ground rules. We have to establish ground rules… And yes, I can switch things around as you grow, and I can bend and lean, but, these are the ground rules and these are the consequences for these actions. And, vice versa - if you do good, you, you get good. If you do bad - you get bad.” So, that’s, I did it, and I hadn’t even thought about that until right now that you asked me again. (laughter) But, I set them down and it’s like, “Okay, you know what? Let’s just be clear about that.” And, I don’t think I would have even thought about it if it wasn’t asking me daily (laughter) about consequences and actions, and, and do I act out in anger, or if I’m tired, or you know?

mother, family 2

This mother also noted how reactivity to the EMA question and response content influenced her parenting behaviors. Another parent reported:

About the [questions], what happened when she did a good job or behaved well this morning? …About half-way through the week, I realized that, I guess I’m really hugging and kissing her when I’m feeling like she behaved well, but I’m not hugging and kissing her and saying, “Oh, you did such a good job.”

mother, family 9

In this example, reactivity to self-monitoring resulted in moving away from awareness to changing her behavior to more actively reinforce her child’s good behavior.

### Sleep

Finally, both children and parents noted that the study made them realize the importance of sleep in their daily routines and self-monitoring:

It helped me ‘cause it, with the questions…on the wake up, like how many hours of sleep, and I’d be tired, and I’d look at the time,...and then I’d be like, “Oh, I only got like seven hours.” And then the day where I’d wake up better, I’d be like, “Oh, I had nine hours,” so that’s better for me.

male child, 14 years, family 5

Another parent gave an example of reflecting on the potential relationship between family conflict and sleep:

Like was there more conflict when I was sleep deprived? (laughter)

mother, family 1

In this example, self-monitoring reactivity first resulted in a strong awareness of sleep and functioning. One mother noted how responding to sleep questions seeded motivation:

I thought the sleep one was a very good question, ‘cause then when I would answer it, I thought, “Ooh, yeah. I better get some more sleep.”

mother, family 11

Overall, these results demonstrate how reactivity to EMA self-monitoring typically begins with increased awareness of behavior, followed by associations with antecedents or consequences. Then, some participants experienced motivation to change, with behavior changes supported by reminder functions of EMA prompts (alarms), accountability to subsequent reporting, and tracking of goal progress and outcomes.

## Discussion

### Principal Findings

The results of this study support the high feasibility and acceptability of using a smartphone EMA by young adolescents and parents for assessing and self-monitoring family daily routines and interactions over 2 weeks, as evidenced by high response rates of 95% and greater and in user-experience interviews. Some participants suggested that a third or fourth week of self-monitoring would further enhance the behavioral changes that they initiated. Some participants also reported preferring fewer surveys each day and fewer questions, particularly when considering a longer duration of self-monitoring beyond 2 to 3 weeks.

Our findings also suggest that smartphone self-monitoring may be a useful tool to support improvement in family functioning through functions of reflection on antecedents and consequences of situations, prompting positive and negative alternatives, seeding goals, and reinforcement by self-tracking for self-correction and self-rewards. These functions are core elements of self-regulation [14-17], which may now be enhanced by smartphone integration into daily routines. Reactivity in self-monitoring has been documented for a wide range of clinically relevant behaviors and may make an adjunctive contribution to intervention efforts [44]. The portability and convenience of smartphone integration into daily routines is creating novel opportunities to reinvigorate research on self-monitoring. Participants in this study reported increased awareness of their family routines, and many also reported behavioral changes in terms of decision making, parental monitoring, quantity and quality of time together, communication, self-regulation of stress and conflict, discipline, and sleep.

Our primary findings also suggest there was a potential indication of ethnic and/or income differences among parents in discussing discipline, rewards, and punishments that may warrant further exploration in future research. However, the small sample size does not warrant inferences as a primary result. White parents, from the higher-income site, seemed to be more comfortable discussing parenting practices with an emphasis on rewards and expectations and de-emphasizing punishments or negative reinforcements. Latino parents, from the low-income site, were more inclined to focus on parenting as a *job* to keep their children *safe* and seemed to be more comfortable discussing consequences or negative reinforcements. Some research has conceptualized that parenting practices and discipline may be moderated by the interaction of parental beliefs and ethnicity [45]. However, the small sample and recruitment sites confound not only ethnicity and income but also neighborhood safety, as the low-income neighborhood is noted for gang violence.

### Limitations

This pilot study had several notable limitations. First, the sample size was small in terms of the number of families. Nonetheless, the overall number of participants is typical and acceptable for a qualitatively focused pilot study focused on user experience and feedback, and saturation of themes was achieved. The sample lacked representation from African American and Asian-American families, lower-income white families, and higher-income Latino families. Second, the family wellness center recruitment sites attracted families primed for motivation to improve their family functioning, as did the recruitment material framing the study as seeking support in developing and testing mobile apps to assess and improve family functioning. Third, our measure/domain selection reflects assumptions for successful parenting and interactions that are prevalent in family assessment tools and evidence-based interventions for risky adolescents, while also including domains reflecting resiliency. Fourth, it is important to note that the steep rise in mobile phone use among children and adolescents has also raised concerns about possible adverse effects such as addictive tendencies, depression, anxiety, sleep disruption, and cyberbullying [46,47]. Fifth, the smartphone app did not employ passive monitoring of smartphone usage, which was a separate module in the Ohmage smartphone app platform and was not used for both privacy and battery power preservation concerns. Notably, the study protocol and app did have an audio-sensing module, which continuously and passively monitored and classified the audio environment for speech versus nonspeech (eg, including discrimination of background audio from televisions) in small snippets of privacy-preserving nonaudio data, but the module failed to function outside of the laboratory because of data not being transferred off the phones quickly enough through a mobile connection (as opposed to Wi-Fi in the lab), causing the phones’ operating system to crash. Only the first study family had Audiosens data, and for only several days before the phones crashed. Since the time of the study in 2013, improvements in smartphone technology, memory, and wireless data speeds would now make this component more feasible, and in fact, potentially advancing classification from speech/nonspeech to emotion detection. Finally, the study was not powered for statistical analyses; the primary aims were feasibility, acceptability, user experience, and preliminary perceived efficacy of smartphone app self-monitoring for assessing and potentially improving family routines.

Future research should evaluate reactivity to EMA and diary self-monitoring as a tool to improve family routines in larger and more diverse samples of families, with statistical power to robustly examine behavioral, symptom, state, and functioning changes. Further research could also consider recruiting families coping with challenges such as chronic illnesses, substance abuse, or conduct problems. Future studies should also include longer follow-up periods to examine the sustainability of self-monitoring and decreasing burden over time and the significance of behavior changes indicated by participants in this study. Furthermore, future studies could also provide empirical and theoretical insight into how sibling relationships can serve as important contexts for individual development and family functioning. Due to participant feedback about frequency and duration of assessments, future studies using frequent assessments for highly transient states or frequent behaviors (eg, every 30 min) should be short in duration (eg, seconds to minutes) to minimize response burden over shorter assessment periods (ie, several days) [20]. Conversely, daily assessments may allow for a longer duration over longer periods of time (eg, weeks to months). Finally, future research assessing family functioning should ensure that assessments are adequately collecting data during times or situations of interest to families [20]. For example, if a study examines parent-child interactions, assessments should only occur when parents and children are together, such as mornings, evenings, and weekends (eg, not when at school/work). Future studies should include larger samples with more diverse and higher-risk populations, longer study durations, the inclusion of passive phone sensors and peripheral biometric devices, and integration with counseling and parenting interventions and programs.

### Conclusions

Due to the increasing ease of implementing EMA and diary self-monitoring via smartphones, practitioners may reconsider using smartphones to enhance psychotherapy, parenting programs, and other counseling modalities. Conversely, researchers using EMA and diaries should examine reactivity more consistently and robustly. Real-time data visualization tools (eg, time trends, correlations, and maps) hold the potential to make self-monitoring more salient and actionable through use in counseling sessions for problem solving, feedback, praise, and goal refinement. Machine learning algorithms also hold promise for detecting patterns and anticipating change points to trigger automated in-the-moment or *just-in-time* interventions. Further research is needed on self-monitoring as a purely self-directed intervention activity and the potential for enhancing therapeutic relationships.

## Appendix

Multimedia Appendix 1 EMA Parent Survey.

Multimedia Appendix 2 EMA Child Survey.

Multimedia Appendix 3 Post Interview Questions.

## Abbreviations

EMA: ecological momentary assessment
UCLA: University of California, Los Angeles

## References

[1] Day RD, Padilla-Walker LM (2009). Mother and father connectedness and involvement during early adolescence. J Fam Psychol.

[2] Dickstein S (2002). Family routines and rituals-the importance of family functioning: comment on the special section. J Fam Psychol.

[3] Friesen M, Woodward L, Horwood L, Fergusson D (2013). Quality of parent-child relations in adolescence and later adult parenting outcomes. Soc Dev.

[4] Segrin C, Woszidlo A, Givertz M, Bauer A, Murphy M (2012). The association between overparenting, parent-child communication, and entitlement and adaptive traits in adult children. J Fam Relat.

[5] Levin KA, Dallago L, Currie C (2011). The association between adolescent life satisfaction, family structure, family affluence and gender differences in parent–child communication. Soc Indic Res.

[6] Brown AM, Fitzgerald MM, Shipman K, Schneider R (2007). Children’s expectations of parent–child communication following interparental conflict: do parents talk to children about conflict?. J Fam Viol.

[7] Repetti RL, Robles TF, Reynolds B (2011). Allostatic processes in the family. Dev Psychopathol.

[8] Sarigiani PA, Heath PA, Camarena PM (2016). The significance of parental depressed mood for young adolescents' emotional and family experiences. J Early Adolescence.

[9] Gerard JM, Krishnakumar A, Buehler C (2016). Marital conflict, parent-child relations, and youth maladjustment. J Fam Issues.

[10] Story LB, Repetti R (2006). Daily occupational stressors and marital behavior. J Fam Psychol.

[11] Larson RW, Richards MH (1994). Family emotions: do young adolescents and their parents experience the same states?. J Res Adolescence.

[12] Sossin K, Birklein S (2006). Nonverbal transmission of stress between parent and young child: considerations and psychotherapeutic implications of a study of affective movement patterns. J Infant Child Adolescent Psychother.

[13] Chung GH, Flook L, Fuligni AJ (2011). Reciprocal associations between family and peer conflict in adolescents' daily lives. Child Dev.

[14] Bamberger KT (2016). The application of intensive longitudinal methods to investigate change: stimulating the field of applied family research. Clin Child Fam Psychol Rev.

[15] Bandura A (1991). Social cognitive theory of self-regulation. Organ Behav Hum Decis Process.

[16] Carver CS (1979). A cybernetic model of self-attention processes. J Pers Soc Psychol.

[17] Kanfer FH (1970). Self-monitoring: methodological limitations and clinical applications. J Consult Clin Psychol.

[18] Kazdin AE (1974). Reactive self-monitoring: the effects of response desirability, goal setting, and feedback. J Consult Clin Psychol.

[19] Shiffman S, Stone AA, Hufford MR (2008). Ecological momentary assessment. Annu Rev Clin Psychol.

[20] Smyth J, Heron K, McHale SM, Amato P, Booth A (2014). Ecological momentary assessment (EMA) in family research. National Symposium on Family Issues: Volume 4. Emerging Methods in Family Research.

[21] Ducharme J, Doyle AB, Markiewicz D (2016). Attachment security with mother and father: associations with adolescents' reports of interpersonal behavior with parents and peers. J Soc Pers Relatsh.

[22] Rusby JC, Westling E, Crowley R, Light JM (2013). Concurrent and predictive associations between early adolescent perceptions of peer affiliates and mood states collected in real time via ecological momentary assessment methodology. Psychol Assess.

[23] Heron KE, Smyth JM (2010). Ecological momentary interventions: incorporating mobile technology into psychosocial and health behaviour treatments. Br J Health Psychol.

[24] Johnson S, Bolstad O (1974). Education Resources Information Center.

[25] Ramanathan N, Alquaddoomi F, Falaki H, George D, Hsieh C, Jenkins J (2012). ohmage: An Open Mobile System for Activity and Experience Sampling. Proceedings of the 6th International Conference on Pervasive Computing Technologies for Healthcare and Workshops.

[26] Alderfer MA, Fiese BH, Gold JI, Cutuli JJ, Holmbeck GN, Goldbeck L, Chambers CT, Abad M, Spetter D, Patterson J (2008). Evidence-based assessment in pediatric psychology: family measures. J Pediatr Psychol.

[27] Bloom BL, Naar S (1994). Self-report measures of family functioning: extensions of a factorial analysis. Fam Process.

[28] Bray JH (1995). Family assessment: current issues in evaluating families. J Fam Relat.

[29] Grebelsky-Lichtman T (2013). Parental patterns of cooperation in parent-child interactions: the relationship between nonverbal and verbal communication. Hum Commun Res.

[30] Crawford JR, Henry JD (2004). The positive and negative affect schedule (PANAS): construct validity, measurement properties and normative data in a large non-clinical sample. Br J Clin Psychol.

[31] Pollak J, Adams P, Gay G (2011). PAM: A Photographic Affect Meter for Frequent, In Situ Measurement of Affect. Proceedings of the SIGCHI Conference on Human Factors in Computing Systems.

[32] Stattin H, Kerr M (2000). Parental monitoring: a reinterpretation. Child Dev.

[33] Kerr M, Stattin H, Burk W (2010). A reinterpretation of parental monitoring in longitudinal perspective. J Res Adolescence.

[34] Elgar FJ, Waschbusch DA, Dadds MR, Sigvaldason N (2006). Development and validation of a short form of the Alabama parenting questionnaire. J Child Fam Stud.

[35] Essau CA, Sasagawa S, Frick PJ (2006). Psychometric properties of the Alabama parenting questionnaire. J Child Fam Stud.

[36] Robin A, Foster S (1989). Negotiating Parent-Adolescent Conflict: A Behavioral-Family Systems Approach.

[37] (2019). Bright Futures: A National Health Promotion Initiative.

[38] Furman W, Buhrmester D (2009). The network of relationships inventory: behavioral systems version. Int J Behav Dev.

[39] Straus MA, Hamby SL, Boney-McCOY S, Sugarman DB (2016). The revised conflict tactics scales (CTS2). J Fam Issues.

[40] Furman W, Buhrmester D (2009). The network of relationships inventory: behavioral systems version. Int J Behav Dev.

[41] Bringhurst D, Watson C, Miller S, Duncan B (2006). The reliability and validity of the Outcome rating scale: a replication Study of a brief clinical measure. J Brief Ther.

[42] Miles M, Huberman A (1994). Qualitative Data Analysis: A Methods Sourcebook.

[43] Ryan GW, Bernard HR (2016). Techniques to identify themes. Field Methods.

[44] Korotitsch WJ, Nelson-Gray RO (1999). An overview of self-monitoring research in assessment and treatment. Psychol Assess.

[45] Pinderhughes EE, Dodge KA, Bates JE, Pettit GS, Zelli A (2000). Discipline responses: influences of parents' socioeconomic status, ethnicity, beliefs about parenting, stress, and cognitive-emotional processes. J Fam Psychol.

[46] Sánchez-Martínez M, Otero A (2009). Factors associated with cell phone use in adolescents in the community of Madrid (Spain). Cyberpsychol Behav.

[47] Thomée A, Härenstam A, Hagberg M (2011). Mobile phone use and stress, sleep disturbances, and symptoms of depression among young adults-a prospective cohort study. BMC Public Health.

