# Supplementary material for: Smartphone Self-Monitoring by Young Adolescents and Parents to Assess and Improve Family Functioning: Qualitative Feasibility Study
Source: JMIR Form Res. 2020 Jun 23;4(6):e15777. doi: 10.2196/15777 (PMC7381003; doi:10.2196/15777)
Supplement: Multimedia Appendix 1 [file formative_v4i6e15777_app1.docx]

**Ecological Momentary Assessment Survey- Parent**

Waking Up Survey

- How many hours of sleep did you get last night? (0-24)

- How well did you sleep last night?

[] –2 - Very bad

[] –1 - Bad

[] 0 - Okay

[] +1 - Good

[] +2 - Very good

- What is your stress level right now?

[] 0 - not at all

[] 1 - a little

[] 2 - somewhat

[] 3 - very

[] 4 - extremely stressed

- PAM How are feeling right now

Morning Survey

- What was your stress level over the past few hours?

[] 0 - not at all

[] 1 - a little

[] 2 - somewhat

[] 3 - very

[] 4 - extremely stressed

- PAM How are feeling right now

- PAM How have you been feeling over the past few hours?

- Did you talk to your child this morning? Check all that apply

[] Yes, in person

[] Yes, by phone

[] Yes, by text

[] Yes, by instant messaging or social media (Facebook)

[] Yes, by video chat

[] No, I did not talk with my child this morning

If yes,

- How much time did you and your child spend together or communicating this morning?

[] None

[] A few minutes

[] 10 to 15 minutes

[] 20 to 30 minutes

[] 1 hour

[] 2 hours

[] 3 hours or more

- How much did you and your child argue, disagree, or get upset with each other?

[] 0 - Not at all

[] 1 - A little

[] 2 - Somewhat

[] 3 - Very much

[] 4 - Extremely

*If “Not at all” selected, skip to “What happened with consequences” questions.

If yes,

- What was the disagreement or conflict about? (check all that apply)

[] Chores / Responsibilities

[] Dressing - clothes, hair

[] School – attendance, grades, homework, behavior

[] Friends

[] Disclosure / Secrecy

[] Money

[] Other (specify: )

- How did you try to resolve this disagreement or conflict? (check all that apply)

[] Threatened to give a punishment or consequence

[] Gave a punishment or consequence

[] Tried to discuss the issue calmly

[] Did discuss the issue calmly

[] Got information to back up your side of things

[] Brought in or tried to bring in someone to help settle things

[] Left the room to cool down

[] Yelled, insulted or swore

[] Refused to talk about it

[] Cried

[] Threw, smashed, hit, or kicked something

- How did your child try to resolve this disagreement or conflict (check all that apply)

[] Tried to discuss the issue calmly

[] Did discuss the issue calmly

[] Got information to back up their side of things

[] Brought in or tried to bring in someone to help settle things

[] Left the room to cool down

[] Yelled, insulted or swore

[] Refused to talk about it

[] Cried

[] Threw, smashed, hit, or kicked something

- What happened with consequences between you and your child this morning? (check all that apply)

[] Nothing happened that deserved a consequence

[] You let your child out of consequences early

[] Your child talked you out of a consequence

[] You were going to give a consequence and then did not do it

[] Giving a consequence depended on your mood (either good or bad)

[] Giving a consequence depended on your energy (either tired or rested)

[] You followed through on consequences you set

- What happened when your child did a good job or behaved well this morning? (check all that apply)

[] You told your child that s/he did a good job

[] You hugged or kissed your child

[] You rewarded or gave your child something extra

[] Nothing, because nothing special happened – didn’t do better or worse than normal

[] Nothing, because your child didn't do a good job or behaved poorly

[] Your child did better than usual or behaved well

[] Your child did worse than usual or didn't behave well

Afternoon Survey

- What was your stress level over the past few hours? (1=not stressed, 5=very stressed)

[] 0 - not at all

[] 1 - a little

[] 2 - somewhat

[] 3 - very

[] 4 - extremely stressed

- PAM How are feeling right now

- PAM How have you been feeling over the past few hours?

- Did you talk to your child this afternoon? Check all that apply

[] Yes, in person

[] Yes, by phone

[] Yes, by text

[] Yes, by instant messaging or social media (Facebook)

[] Yes, by video chat

[] No, I did not talk with my child this afternoon

If yes,

- How much time did you and your child spend together or communicating this afternoon?

[] None

[] A few minutes

[] 10 to 15 minutes

[] 20 to 30 minutes

[] 1 hour

[] 2 hours

[] 3 hours or more

- How much did you and your child argue, disagree, or get upset with each other?

[] 0 - Not at all

[] 1 - A little

[] 2 - Somewhat

[] 3 - Very much

[] 4 - Extremely

*If “Not at all” selected, skip to “What happened with consequences” questions.

If yes,

- What was the disagreement or conflict about? (check all that apply)

[] Chores / Responsibilities

[] Dressing - clothes, hair

[] School – attendance, grades, homework, behavior

[] Friends

[] Disclosure / Secrecy

[] Money

[] Other (specify: )

- How did you try to resolve this disagreement or conflict? (check all that apply)

[] Threatened to give a punishment or consequence

[] Gave a punishment or consequence

[] Tried to discuss the issue calmly

[] Did discuss the issue calmly

[] Got information to back up your side of things

[] Brought in or tried to bring in someone to help settle things

[] Left the room to cool down

[] Yelled, insulted or swore

[] Refused to talk about it

[] Cried

[] Threw, smashed, hit, or kicked something

- How did your child try to resolve this disagreement or conflict (check all that apply)

[] Tried to discuss the issue calmly

[] Did discuss the issue calmly

[] Got information to back up their side of things

[] Brought in or tried to bring in someone to help settle things

[] Left the room to cool down

[] Yelled, insulted or swore

[] Refused to talk about it

[] Cried

[] Threw, smashed, hit, or kicked something

- What happened with consequences between you and your child this afternoon? (check all that apply)

[] Nothing happened that deserved a consequence

[] You let your child out of consequences early

[] Your child talked you out of a consequence

[] You were going to give a consequence and then did not do it

[] Giving a consequence depended on your mood (either good or bad)

[] Giving a consequence depended on your energy (either tired or rested)

[] You followed through on consequences you set

- What happened when your child did a good job or behaved well this afternoon? (check all that apply)

[] You told your child that s/he did a good job

[] You hugged or kissed your child

[] You rewarded or gave your child something extra

[] Nothing, because nothing special happened – didn’t do better or worse than normal

[] Nothing, because your child didn't do a good job or behaved poorly

[] Your child did better than usual or behaved well

[] Your child did worse than usual or didn't behave well

Evening Survey

- What was your stress level over the past few hours?

[] 0 - not at all

[] 1 - a little

[] 2 - somewhat

[] 3 - very

[] 4 - extremely stressed

- PAM How are feeling right now

- PAM How have you been feeling over the past few hours?

- Did you talk to your child this evening? Check all that apply

[] Yes, in person

[] Yes, by phone

[] Yes, by text

[] Yes, by instant messaging or social media (Facebook)

[] Yes, by video chat

[] No, I did not talk with my child this evening

*If “No, I did not talk with my child this evening” selected, skip to “Today you knew…” questions.

If yes,

- How much time did you and your child spend together or communicating this evening?

[] None

[] A few minutes

[] 10 to 15 minutes

[] 20 to 30 minutes

[] 1 hour

[] 2 hours

[] 3 hours or more

- How much did you and your child argue, disagree, or get upset with each other?

[] 0 - Not at all

[] 1 - A little

[] 2 - Somewhat

[] 3 - Very much

[] 4 - Extremely

*If “Not at all” selected, skip to “What happened with consequences” questions.

If yes,

- What was the disagreement or conflict about? (check all that apply)

[] Chores / Responsibilities

[] Dressing - clothes, hair

[] School – attendance, grades, homework, behavior

[] Friends

[] Disclosure / Secrecy

[] Money

[] Other (specify: )

- How did you try to resolve this disagreement or conflict? (check all that apply)

[] Threatened to give a punishment or consequence

[] Gave you a punishment or consequence

[] Tried to discuss the issue calmly

[] Did discuss the issue calmly

[] Got information to back up your side of things

[] Brought in or tried to bring in someone to help settle things

[] Left the room to cool down

[] Yelled, insulted or swore

[] Refused to talk about it

[] Cried

[] Threw, smashed, hit, or kicked something

- How did your child try to resolve this disagreement or conflict (check all that apply)

[] Tried to discuss the issue calmly

[] Did discuss the issue calmly

[] Got information to back up their side of things

[] Brought in or tried to bring in someone to help settle things

[] Left the room to cool down

[] Yelled, insulted or swore

[] Refused to talk about it

[] Cried

[] Threw, smashed, hit, or kicked something

- What happened with consequences between you and your child this evening? (check all that apply)

[] Nothing happened that deserved a consequence

[] You let your child out of consequences early

[] Your child talked you out of a consequence

[] You were going to give a consequence and then did not do it

[] Giving a consequence depended on your mood (either good or bad)

[] Giving a consequence depended on your energy (either tired or rested)

[] You followed through on consequences you set

- What happened when your child did a good job or behaved well this evening? (check all that apply)

[] You told your child that s/he did a good job

[] You hugged or kissed your child

[] You rewarded or gave your child something extra

[] Nothing, because nothing special happened – didn’t do better or worse than normal

[] Nothing, because your child didn't do a good job or behaved poorly

[] Your child did better than usual or behaved well

[] Your child did worse than usual or didn't behave well

- What interaction did you have with your child today (check all that applied)

[] Took to a special activity

[] Played games or did other fun activities

[] Helped with a special activity (like sports or scouts)

[] Asked about day (at school or activities)

[] Helped with homework or a project

[] Watched TV or a movie together

[] Ate a meal together

[] Touched bases to check-in

[] Other (specify: )

- Today, you knew. (check all that apply)

[] about how your child was doing with their friends

[] what happened or how your child is doing in school or activities

[] what your child did during free time

[] what homework, papers, or tests your child had

[] where your child went and what your child did after school

[] what your child watched on TV or the internet (e.g., YouTube)

[] what your child did online (video games, Facebook, IM, websites)

[] who your child talked to (including online, phone, text)

- Today, your child told you, without you asking. (check all that apply)

[] about his/her friends

[] what happened or how your child is doing in school or activities

[] what your child did during free time

[] what homework, papers, or tests your child had

[] where your child went and what your child did after school

[] what your child watched on TV or the internet (e.g., YouTube)

[] what your child did online (video games, Facebook, IM, websites)

[] who your child talked to (including online, phone, text)

- Today, you asked your child. (check all that apply)

[] about your child's friends

[] what happened or how your child is doing in school or activities

[] what your child did during free time

[] what homework, papers, or tests your child had

[] where your child went and what your child did after school

[] what your child watched on TV or the internet (e.g., YouTube)

[] what your child did online (video games, Facebook, IM, websites)

[] who your child talked to (including online, phone, text)

- How much did you and your child spend free time together or d0 something fun together today?

[] 0 - Not at all

[] 1 - A little

[] 2 - Somewhat

[] 3 - Very much

[] 4 - Extremely

- How much did you and your child argue, disagree, or get upset with each other today?

[] 0 - Not at all

[] 1 - A little

[] 2 - Somewhat

[] 3 - Very much

[] 4 - Extremely

- How much did you and your child say mean or harsh things, criticize, or put each other down today?

[] 0 - Not at all

[] 1 - A little

[] 2 - Somewhat

[] 3 - Very much

[] 4 - Extremely

- How much did you and your child nag, get annoyed, or get on each other's nerves today?

[] 0 - Not at all

[] 1 - A little

[] 2 - Somewhat

[] 3 - Very much

[] 4 - Extremely

- Who did you eat dinner with today? (check all that apply)

[] Spouse/Partner

[] Child

[] Child's Brother(s)

[] Child's Sister(s)

[] Alone

[] Other relatives

[] Other people

[] Did not eat dinner

If not "Did not eat dinner",

- Were you doing anything else during dinner? (like watching TV, texting, playing video games)

[] Yes

[] No

- How are you doing overall today? (1=worst, 10=best)

- How are things in your family today? (1=worst, 10=best)

- How is your child doing at school or in other activities today? (1=worst, 10=best)

- How is everything going for your child today? (1=worst, 10=best)
