# Supplementary material for: Smartphone Self-Monitoring by Young Adolescents and Parents to Assess and Improve Family Functioning: Qualitative Feasibility Study
Source: JMIR Form Res. 2020 Jun 23;4(6):e15777. doi: 10.2196/15777 (PMC7381003; doi:10.2196/15777)
Supplement: Multimedia Appendix 2 [file formative_v4i6e15777_app2.docx]

**Ecological Momentary Assessment Survey- Child**

Waking Up Survey

- How many hours of sleep did you get last night? (0-24)

- How well did you sleep last night?

[] –2 - Very bad

[] –1 - Bad

[] 0 - Okay

[] +1 - Good

[] +2 - Very good

- What is your stress level right now?

[] 0 - not at all

[] 1 - a little

[] 2 - somewhat

[] 3 - very

[] 4 - extremely stressed

- PAM How are feeling right now

Morning Survey

- What was your stress level over the past few hours?

[] 0 - not at all

[] 1 - a little

[] 2 - somewhat

[] 3 - very

[] 4 - extremely stressed

- PAM How are feeling right now

- PAM How have you been feeling over the past few hours?

- Did you talk to your parent this morning? Check all that apply

[] Yes, in person

[] Yes, by phone

[] Yes, by text

[] Yes, by instant messaging or social media (Facebook)

[] Yes, by video chat

[] No, I did not talk with my parent this morning

If yes,

- How much time did you and your parent spend together or communicating this morning?

[] None

[] A few minutes

[] 10 to 15 minutes

[] 20 to 30 minutes

[] 1 hour

[] 2 hours

[] 3 hours or more

- How much did you and your parent argue, disagree, or get upset with each other?

[] 0 - Not at all

[] 1 - A little

[] 2 - Somewhat

[] 3 - Very much

[] 4 - Extremely

*If “Not at all” selected, skip to “What happened with consequences” questions.

If yes,

- What was the disagreement or conflict about? (check all that apply)

[] Chores / Responsibilities

[] Dressing - clothes, hair

[] School – attendance, grades, homework, behavior

[] Friends

[] Disclosure / Secrecy

[] Money

[] Other (specify: )

- How did your parent try to resolve this disagreement or conflict? (check all that apply)

[] Threatened to give a punishment or consequence

[] Gave you a punishment or consequence

[] Tried to discuss the issue calmly

[] Did discuss the issue calmly

[] Got information to back up their side of things

[] Brought in or tried to bring in someone to help settle things

[] Left the room to cool down

[] Yelled, insulted or swore

[] Refused to talk about it

[] Cried

[] Threw, smashed, hit, or kicked something

- How did you try to resolve this disagreement or conflict (check all that apply)

[] Tried to discuss the issue calmly

[] Did discuss the issue calmly

[] Got information to back up my side of things

[] Brought in or tried to bring in someone to help settle things

[] Left the room to cool down

[] Yelled, insulted or swore at your parent

[] Refused to talk about it

[] Cried

[] Threw, smashed, hit, or kicked something

- What happened with consequences between you and your parent this morning? (check all that apply)

[] Nothing happened that deserved a consequence

[] Your parent let you out of consequence early

[] You talked your parent out of a consequence

[] Your parent was going to give a consequence and then did not do it

[] Giving a consequence depended on your parent’s mood (either good or bad)

[] Giving a consequence depended on your parent’s energy (either tired or rested)

[] Your parent followed through on consequences they set

- What happened when you did a good job or behaved well this morning? (check all that apply)

[] Your parent told you that you did a good job

[] Your parent hugged or kissed you

[] Your parent rewarded or gave you something extra

[] Nothing, because nothing special happened – didn’t do better or worse than normal

[] Nothing, because you didn't do a good job or behaved poorly

[] You did better than usual or behaved well

[] You did worse than usual or didn't behave well

Afternoon Survey

- What was your stress level over the past few hours?

[] 0 - not at all

[] 1 - a little

[] 2 - somewhat

[] 3 - very

[] 4 - extremely stressed

- PAM How are feeling right now

- PAM How have you been feeling over the past few hours?

- Did you talk to your parent this afternoon? Check all that apply

[] Yes, in person

[] Yes, by phone

[] Yes, by text

[] Yes, by instant messaging or social media (Facebook)

[] Yes, by video chat

[] No, I did not talk with my parent this afternoon

If yes,

- How much time did you and your parent spend together or communicating this morning?

[] None

[] A few minutes

[] 10 to 15 minutes

[] 20 to 30 minutes

[] 1 hour

[] 2 hours

[] 3 hours or more

- How much did you and your parent argue, disagree, or get upset with each other?

[] 0 - Not at all

[] 1 - A little

[] 2 - Somewhat

[] 3 - Very much

[] 4 - Extremely

If yes,

- What was the disagreement or conflict about? (check all that apply)

[] Chores / Responsibilities

[] Dressing - clothes, hair

[] School – attendance, grades, homework, behavior

[] Friends

[] Disclosure / Secrecy

[] Money

[] Other (specify: )

- How did your parent try to resolve this disagreement or conflict? (check all that apply)

[] Threatened to give a punishment or consequence

[] Gave you a punishment or consequence

[] Tried to discuss the issue calmly

[] Did discuss the issue calmly

[] Got information to back up their side of things

[] Brought in or tried to bring in someone to help settle things

[] Left the room to cool down

[] Yelled, insulted or swore

[] Refused to talk about it

[] Cried

[] Threw, smashed, hit, or kicked something

- How did you try to resolve this disagreement or conflict (check all that apply)

[] Tried to discuss the issue calmly

[] Did discuss the issue calmly

[] Got information to back up my side of things

[] Brought in or tried to bring in someone to help settle things

[] Left the room to cool down

[] Yelled, insulted or swore at your parent

[] Refused to talk about it

[] Cried

[] Threw, smashed, hit, or kicked something

- What happened with consequences between you and your parent this morning? (check all that apply)

[] Nothing happened that deserved a consequence

[] Your parent let you out of consequence early

[] You talked your parent out of a consequence

[] Your parent was going to give a consequence and then did not do it

[] Giving a consequence depended on your parent’s mood (either good or bad)

[] Giving a consequence depended on your parent’s energy (either tired or rested)

[] Your parent followed through on consequences they set

- What happened when you did a good job or behaved well this afternoon? (check all that apply)

[] Your parent told you that you did a good job

[] Your parent hugged or kissed you

[] Your parent rewarded or gave you something extra

[] Nothing, because nothing special happened – didn’t do better or worse than normal

[] Nothing, because you didn't do a good job or behaved poorly

[] You did better than usual or behaved well

[] You did worse than usual or didn't behave well

Evening Survey

- What was your stress level over the past few hours?

[] 0 - not at all

[] 1 - a little

[] 2 - somewhat

[] 3 - very

[] 4 - extremely stressed

- PAM How are feeling right now

- PAM How have you been feeling over the past few hours?

- Did you talk to your parent this evening? Check all that apply

[] Yes, in person

[] Yes, by phone

[] Yes, by text

[] Yes, by instant messaging or social media (Facebook)

[] Yes, by video chat

[] No, I did not talk with my parent this evening

If yes,

- How much time did you and your parent spend together or communicating this evening?

[] None

[] A few minutes

[] 10 to 15 minutes

[] 20 to 30 minutes

[] 1 hour

[] 2 hours

[] 3 hours or more

- How much did you and your parent argue, disagree, or get upset with each other?

[] 0 - Not at all

[] 1 - A little

[] 2 - Somewhat

[] 3 - Very much

[] 4 - Extremely

*If “Not at all” selected, skip to “What happened with consequences” questions.

If yes,

- What was the disagreement or conflict about? (check all that apply)

[] Chores / Responsibilities

[] Dressing - clothes, hair

[] School – attendance, grades, homework, behavior

[] Friends

[] Disclosure / Secrecy

[] Money

[] Other (specify: )

- How did your parent try to resolve this disagreement or conflict? (check all that apply)

[] Threatened to give a punishment or consequence

[] Gave you a punishment or consequence

[] Tried to discuss the issue calmly

[] Did discuss the issue calmly

[] Got information to back up their side of things

[] Brought in or tried to bring in someone to help settle things

[] Left the room to cool down

[] Yelled, insulted or swore

[] Refused to talk about it

[] Cried

[] Threw, smashed, hit, or kicked something

- How did you try to resolve this disagreement or conflict (check all that apply)

[] Tried to discuss the issue calmly

[] Did discuss the issue calmly

[] Got information to back up my side of things

[] Brought in or tried to bring in someone to help settle things

[] Left the room to cool down

[] Yelled, insulted or swore at your parent

[] Refused to talk about it

[] Cried

[] Threw, smashed, hit, or kicked something

- What happened with consequences between you and your parent this evening? (check all that apply)

[] Nothing happened that deserved a consequence

[] Your parent let you out of consequence early

[] You talked your parent out of a consequence

[] Your parent was going to give a consequence and then did not do it

[] Giving a consequence depended on your parent’s mood (either good or bad)

[] Giving a consequence depended on your parent’s energy (either tired or rested)

[] Your parent followed through on consequences they set

- What happened when you did a good job or behaved well this evening? (check all that apply)

[] Your parent told you that you did a good job

[] Your parent hugged or kissed you

[] Your parent rewarded or gave you something extra

[] Nothing, because nothing special happened – didn’t do better or worse than normal

[] Nothing, because you didn't do a good job or behaved poorly

[] You did better than usual or behaved well

[] You did worse than usual or didn't behave well

- What interaction did your parent have with you today (check all that applied)

[] Took to a special activity

[] Played games or did other fun activities with you

[] Helped you with a special activity (like sports or scouts)

[] Asked about your day (at school or activities)

[] Helped you with homework or a project

[] Watched TV or a movie together

[] Ate a meal together

[] Touched bases to check in

[] Other (specify: )

- Today, your parent knew. (check all that apply)

[] about how you are doing with your friends

[] what happened or how you are doing in school or activities

[] what you did during your free time

[] what homework, papers, or tests you had

[] where you went and what you did after school

[] what you watched on TV or the internet (e.g., YouTube)

[] what you did online (video games, Facebook, IM, websites)

[] who you talked to (including online, phone, text)

- Today, you told your parent, without them asking you. (check all that apply)

[] about your friends

[] what happened or how you are doing in school or activities

[] what you did during your free time

[] what homework, papers, or tests you had

[] where you went and what you did after school

[] what you watched on TV or the internet (e.g., YouTube)

[] what you did online (video games, Facebook, IM, websites)

[] who you talked to (including online, phone, text)

- Today, your parent asked you. (check all that apply)

[] about your friends

[] what happened or how you are doing at school or activities

[] what you did during your free time

[] what homework, papers, or tests you had

[] where you went and what you did after school

[] what you watched on TV or the internet (e.g., YouTube)

[] what you did online (video games, Facebook, IM, websites)

[] who you talked to (including online, phone, text)

- How much did you and your parent spend free time together or do something fun together today?

[] 0 - Not at all

[] 1 - A little

[] 2 - Somewhat

[] 3 - Very much

[] 4 – Extremely

- How much did you and your parent argue, disagree, or get upset with each other today?

[] 0 - Not at all

[] 1 - A little

[] 2 - Somewhat

[] 3 - Very much

[] 4 –Extremely

- How much did you and your parent say mean or harsh things, criticize, or put each other down today?

[] 0 - Not at all

[] 1 - A little

[] 2 - Somewhat

[] 3 - Very much

[] 4 – Extremely

- How much did you and your parent nag, get annoyed, or get on each other's nerves today?

[] 0 - Not at all

[] 1 - A little

[] 2 - Somewhat

[] 3 - Very much

[] 4 – Extremely

- Who did you eat dinner with today? (check all that apply)

[] Mother

[] Father

[] Brother(s)

[] Sister(s)

[] Alone

[] Other relatives

[] Other people

[] Did not eat dinner

If not "Did not eat dinner",

- Were you doing anything else during dinner? (like watching TV, texting, playing video games)

[] Yes

[] No

- How are you doing overall today? (1=worst, 10=best)

- How are things in your family today? (1=worst, 10=best)

- How are you doing at school or in other activities today? (1=worst, 10=best)

- How is everything going today? (1=worst, 10=best)
