# Supplementary material for: Smartphone Self-Monitoring by Young Adolescents and Parents to Assess and Improve Family Functioning: Qualitative Feasibility Study
Source: JMIR Form Res. 2020 Jun 23;4(6):e15777. doi: 10.2196/15777 (PMC7381003; doi:10.2196/15777)
Supplement: Multimedia Appendix 3 [file formative_v4i6e15777_app3.docx]

**Questions for Open-Ended Interview**

Can you describe your experiences in this study?

Probe: Can you describe some experiences you enjoyed?
Probe: can you describe some experiences that you found frustrating or annoying or even just boring?

What was most useful or helpful to you in the study?  Explain.

What did you find least helpful or useful in the study? Explain.

Are there any parts of this study you would recommend we change or remove from any future research like this?  Why would you recommend this change?
